# Toward efficient screening systems for sunflower drought tolerance: a comparative and molecular perspective

**DOI:** 10.3389/fbioe.2025.1735259

**Published:** 2026-02-19

**Authors:** Malin Alf, Alessia Ronchi, Aleksandra Radanović, Florian Schilling, Kimon Ionas, Boško Dedić, Srđan Bursać, Siniša Jocić, Zeena D. Costa Rodriguez, Serena Varotto, Dragana Miladinović, Renate Horn

**Affiliations:** 1 Department of Plant Genetics, Institute of Biological Sciences, University of Rostock, Rostock, Germany; 2 Department of Agronomy, Food, Natural resources, Animals and Environment, University of Padova, Padua, Italy; 3 Institute of Field and Vegetable Crops, National Institute of the Republic of Serbia, Novi Sad, Serbia

**Keywords:** abscisic acid, climate-resilient sunflower, drought stress, *in vitro* screening, pot trials, transcriptome

## Abstract

The observed longer and more frequent periods of drought in recent years have drawn more focus on efficient breeding techniques for drought-tolerant crops. Several systems have been used to simulate drought conditions in order to select drought-tolerant lines. However, little is known about the comparability of different screening methods. We compared four different systems in terms of the information provided on drought tolerance, namely, two *in vitro* systems (liquid and solid medium applying polyethylene glycol as the drought simulant), and two soil-based pot trials were conducted either in a green house or in a climate chamber. Two sunflower inbred lines differing in drought tolerance (AB-OR-8 and DF-AB-2) were characterized at the seedling stage using physiological and morphological parameters of shoots and roots. Transcriptomic analysis (RNA-seq) and quantitative RT-PCR were performed to compare drought responses and stress levels applied by the different systems. In an assessment of all four screening methods, the expressions of five of six selected genes in the ABA signaling pathway were differentially upregulated during drought, while one of them was downregulated. These genes represent valuable indicators of the stress level applied by a drought-screening method. The advantages and disadvantages of different drought-simulating systems were evaluated and combined with next-generation sequencing data to identify the most efficient selection approach for assessing drought tolerance in sunflower. Overall, all systems enabled effective pre-screening of the breeding material for drought tolerance, where the decision for a system depends on experimental goals and resource availability. If the goal is to maximize the molecular resolution of drought stress, the *in vitro* liquid system is the most effective.

## 1 Introduction

Sunflower (*Helianthus annuus* L.) is a globally cultivated oilseed crop known for its adaptability to diverse agro-climatic conditions. However, its productivity is increasingly threatened by drought stress, particularly in regions experiencing erratic rainfall and rising temperatures. Drought stress is recognized as one of the most significant abiotic stresses, strongly affecting plant growth and development (Gupta et al., 2020; Haghpanah et al., 2024). Plants have developed a range of adaptive strategies to cope with water deficiency, including escape, avoidance, and drought tolerance, to prevent cellular dehydration (Hurtado et al., 2025). These responses are supported by a suite of morphological, physiological, and molecular adjustments, such as changes in plant morphology and structure, stomatal closure, activation of drought-responsive genes, modulation of hormone pathways, and accumulation of compatible solutes (Krannich et al., 2015; Yang et al., 2021). However, adaptations of plants to water limitations have been evolutionary developed to guarantee the survival of plants, which in most cases diverges from the aim of maintaining a normal yield under drought conditions, which would be defined as drought tolerance for breeding purposes.

To more effectively investigate plant responses to drought stress, researchers have established a range of drought stress-simulating systems such as field trials that are space- and time-consuming. These methods can be divided into three categories based on their fundamental setup, namely, soil-based, aqueous culture-based, or agar-based systems (Osmolovskaya et al., 2018). All these different experimental methods aim to reduce water potential in the medium surrounding the plant roots, thus simulating drought stress conditions. However, each method has its own specific limitations and is only suited to address distinct scientific questions. Thus, carefully considering the advantages and disadvantages of each method is essential when planning drought simulating experiments.

*In vitro* systems are well-suited for detailed molecular and genetic analyses due to their precise control over water availability at the cellular level, though they lack the complexity of natural soil–root interactions. Another limitation is that these systems require the use of osmotically active substances such as polyethylene glycol (PEG), which limits oxygen diffusion to roots and impairs the ion uptake (Mexal et al., 1975; Yeo and Flowers, 1984), or sugars such as mannitol or sorbitol (Wellpott et al., 2024), which are taken up by the plants.

Greenhouse experiments provide a compromise between control and field conditions, enabling the study of whole plants in soil or soil-like substrates under semi-controlled conditions of temperature, humidity, and light. Controlled environment chambers, including walk-in chambers, on the other hand, provide enhanced environmental regulation, enabling more sophisticated simulation of drought patterns in more controlled conditions and facilitating integrative studies of gene expression alongside morphological and physiological changes. Selecting the appropriate drought simulation method is not merely a technical choice—it fundamentally shapes the scope, resolution, and relevance of the biological insights gained. A key part of this challenge lies in choosing which traits to measure and how frequently, with attention to their spatiotemporal dynamics. There is no one-size-fits-all approach to studying plant stress responses; both trait selection and data interpretation must be aligned with the specific goals and context of the experiment (Moshelion et al., 2024). Guidelines regarding the experimental design to avoid biased results were developed. Following them will improve the reproducibility of the results of a complex trait such as drought tolerance (Moshelion et al., 2024).

Drought experiments in sunflower have been performed as field trials (Gul et al., 2021; Moschen et al., 2017), pot trials (Janzen et al., 2023; Sarazin et al., 2017; Shen et al., 2023), and as *in vitro* systems (Fulda et al., 2011; Li et al., 2021; Liang et al., 2017; Vassilevska-Ivanova et al., 2014). Even though different systems have been successfully applied in sunflower to investigate drought stress, the results are not directly comparable because different genotypes were used in all the cases, making it difficult to determine whether the differences are due to the different genotypes or differences in the systems used to induce the drought response. In this study, we offer a direct comparison between four different drought-inducing systems using the same two genotypes that vary in drought tolerance.

At the molecular level, sunflower responds to drought through the activation of drought-responsive genes involved in signal transduction, transcriptional regulation, and stress protection. Analyses of differentially expressed genes (DEGs) have shown that, in sunflower, drought stress generally results in more genes being downregulated than upregulated (Shen et al., 2023). However, several transcription factors (TFs), including members of the bHLH, AP2/ERF-ERF, MYB, C2H2, and NAC families, which are specifically induced in response to drought, play an important role in regulating downstream stress-related genes (Li et al., 2021; Negussu et al., 2023; Shinozaki and Yamaguchi-Shinozaki, 2007; Wu et al., 2022a). These target genes are involved in osmolyte synthesis, detoxification of reactive oxygen species (ROS), and late embryogenesis abundant (LEA) proteins, which collectively enhance cellular protection and provide water retention under stress (Shen et al., 2023; Usman et al., 2025). Transcriptomic analyses have also shown substantial differences in gene expression profiles between drought-tolerant and drought-susceptible sunflower varieties, suggesting genotype-specific regulatory mechanisms that contribute to differential stress adaptation (Hussain et al., 2017; Tran et al., 2024; Wu et al., 2022b). In addition, several genes of ABA biosynthesis and signaling have been associated with drought tolerance in sunflower (Shi et al., 2024).

Drought stress is primarily perceived by the roots (Kalra et al., 2024). Calcium channels are activated in response to water deficit, leading to Ca^2+^ influx into the cytosol (Sanders et al., 1999; Huang et al., 2017). Ca^2+^ activates the biosynthesis of abscisic acid (ABA) through a signaling cascade. ABA then plays an essential role in mediating specific drought stress responses such as stomatal closure and modification of root architecture to minimize water loss and maximize water uptake (Aslam et al., 2022). ABA is synthesized *via* the carotenoid pathway within the plastids. C40 precursors are converted *via* epoxidation and isomerization through several steps. The key enzyme of ABA biosynthesis is 9-cis-epoxycarotenoid dioxygenase (NCED), which catalyzes the formation of xanthoxin, the direct precursor of ABA (Tan et al., 1997; Seo and Koshiba, 2002). ABA activates the ABA signaling pathway, leading to stomatal closure to prevent water loss (Bharath et al., 2021; Wei et al., 2025). ABA in the cytosol binds to pyrabactin resistance 1/pyrabactin resistance 1-like/regulatory component of ABA receptors (PYR/PYL/RCAR), which leads to the inhibition of protein phosphatase 2C (PP2C) (Ma et al., 2009; Park et al., 2009). While PP2C dephosphorylates and, therefore, inactivates SnRK2 kinases in the absence of ABA, the presence of ABA inhibits the phosphatase activity, and SnRK2, on its part, activates downstream targets. These targets include guard cells, leading directly to stomatal closure, and transcription factors influencing further downstream effects (Gupta et al., 2020). These transcription factors include bZIP, MYB, NAC, WRKY, DREB, and ERF (Nakashima et al., 2014). All of these regulate drought responses by regulating the expressions of drought-responsive genes that help in osmoprotection and ROS scavenging (Singh et al., 2015).

In our study, we compared four different screening systems to apply drought stress, namely, two *in vitro* systems (liquid and solid) and two soil-based pot trials (greenhouse and climate chamber), and we point out the advantages and disadvantages of the different systems in order to enable other researchers to make advanced decisions regarding which system would be the best for their purpose. The unique feature is that all experiments were performed with the same two sunflower genotypes, AB-OR-8 and DF-AB-2, which differ in drought tolerance. The morphological and physiological traits of sunflower genotypes under control and drought stress conditions were evaluated. In addition, RNA-seq analyses of control and drought stress conditions in the different systems were performed for AB-OR-8 and DF-AB-2. We focused on the expression analyses of ABA biosynthesis and signaling as the involved genes are ideal candidates to evaluate the drought stress level of a system. Quantitative RT-PCR for six selected genes of ABA signaling allowed an estimation of the drought stress level applied in each of the systems.

## 2 Materials and methods

### 2.1 Plant material

The same two sunflower lines, AB-OR-8 (drought-sensitive) and DF-AB-2 (drought-tolerant), were used for all laboratory experiments. Seeds for these two genotypes were provided by the Institute of Field and Vegetable Crops in Novi Sad, Serbia.

### 2.2 *In vitro* liquid MS-medium

For the *in vitro* experiments in liquid MS-medium (Murashige and Skoog, 1962), 120 seeds were surface-sterilized by incubating them in 4% NaClO with a few drops of Tween 20 for 10 min at 37 °C. After surface sterilization, the seeds were washed thrice for 10 min at 37 °C with distilled and autoclaved water. Surface-sterilized seeds were stored in a sterile Petri dish for further use. Media for the control and stress conditions were prepared using liquid MS-medium (M0221, Duchefa, Haarlem, Netherlands) without any additions. Polyethylene glycol 6000 (PEG 6000) (Carl Roth GmbH + Co. KG, Karlsruhe, Germany) was applied as a drought stress-simulant. For both the control (MS0, 0% PEG 6000, 0.02 MPa) and drought stress (MS4, 15% PEG 6000, -0,364 MPa) media, pH was adjusted to 5.8–5.9. For the MS4 medium, the pH was adjusted before and after adding PEG 6000. All media were autoclaved for 20 min at 121 °C.

For the *in vitro* experiment, WECK jars (“Sturzglas,” J. Weck GmbH u. Co. KG, Wehr-Öflingen, Germany) of three sizes (370, 580, and 825 mL) and lids were sterilized (160 °C, 4 h). All other equipment was autoclaved for 20 min at 121 °C. In each jar, 10 seeds were placed on a mesh (2 mm pore size) on top of a metal frame (1 cm height) at the bottom of the 580-mL jar. The MS0 medium was filled up to the mesh (50 mL) so that later, the seeds touched the medium but did not float. The surface-sterilized seeds were arranged alternately with the tip out- and inward. Each biological replication consisted of a total of six jars (*n*= 60 seeds), both for the control and for the drought stress treatment of each genotype. Three of these jars were used for phenotypic analysis, and three were used for RNA analysis. Leukopor tape (BSN Medical GmbH, Hamburg, Germany) was used to close the lids and enable gas exchange without posing a risk of contamination. The seeds were germinated in a climate chamber (21 °C, 16 h light/8 h dark, 150 μE/m^2^s). After 3 days, the seedlings were transferred to bigger jars to give the shoots and roots more space to grow. A higher support frame (3 cm) was placed in a larger jar (825 mL), and the mesh with the seedlings was placed on top using tweezers. The jars were filled with either the control or stress medium (150 mL). An extra jar (370 mL) was used as a lid and secured with Leukopor tape. The jars were placed in the climate chamber in a randomized way for 11 more days (21 °C, 16 h light/8 h dark, 150 μE/m^2^s).

At the end of the experiment, after 14 days, all seedlings (apart from seedlings that did not develop a root ≥ 3 mm) from the three jars were assessed phenotypically (*n* ≥ 40 per genotype x treatment). For the phenotypic evaluation of the *in vitro* cultivated seedlings, the shoots in total (above ground fresh weight), cotyledons, and primary leaves were individually weighed. Lengths of hypocotyls were measured, and the primary leaves and cotyledons were placed under a glass plate on top of a lighting table and photographed for leaf area measurements. The leaf area was measured using ImageJ software (ImageJ 1.53t). For assessment of root parameters, they were taken out of the medium, dried on a paper towel, and weighed. For further analysis, the roots were stored in 50% ethanol solution. The root parameters were analyzed with WinRHIZO™ (Epson STD4800, WinRHIZO Pro, Regent Instruments, Québec, Canada) and ImageJ software (ImageJ 1.53t). The root-to-shoot ratio was calculated using the root fresh weight and the above-ground fresh weight. All data were statistically evaluated and visualized using R (version 4.5.1). Three individual rounds of experiments (independent biological replications) were conducted.

In addition, samples for RNA extraction were taken from the three remaining jars of each biological replication. For each biological replication, all primary leaves of each genotype x treatment combination (plants *n* ≥ 40) were pooled to obtain enough material for RNA isolation to perform RNA-seq and RT-qPCR. Samples were immediately frozen in liquid nitrogen and stored at – 80 °C for further use.

### 2.3 *In vitro* solid MS-medium

A total of 250 seeds of each genotype were first washed with Tween20 and then thoroughly washed with distilled water (dH_2_O). Then, these seeds were surface-sterilized by incubating them in 14% NaClO for 15 min. After surface sterilization, the seeds were washed five times with dH_2_O and placed in sterile Petri dishes with filter paper to which 4 mL of MS-medium (Murashige and Skoog, 1962) without sucrose and agar was added. Following 1 day of incubation, an additional 2 mL of MS-medium was added to ensure sufficient moisture for seed germination. Subsequently, after 2 days of incubation at 25 °C, seeds were peeled and transferred to the appropriate medium (control and drought) in Erlenmeyer flasks. Three seeds were placed in each flask. In total, 14 flasks per genotype x treatment combination were set. In total, 84 seeds of each examined sunflower line were used in the experiment. For both the control and drought treatments, the MS medium adjusted to pH 5.8 was supplemented with 20% sucrose. The control medium contained 4% phytagel (Sigma Chemical Co.), while the drought medium contained 3.7% phytagel and was supplemented with 4% filter-sterilized polyethylene glycol 6000 (PEG 6000; VWR Chemicals, Belgium). The phytagel concentration in the drought medium was slightly decreased to facilitate PEG–phytagel interaction, which otherwise increases the medium rigidity. Flasks with seedlings representing both control and drought stress conditions were cultivated in a growth chamber at 25 °C and photoperiod of 16 h day and 8 h night for 12 days. From a subset of flasks (10 flasks), primary leaves were harvested from each 14-day-old seedling. Leaves were flash-frozen in liquid nitrogen and deep freezed at −80 °C for storage. For RNA isolation, three biological replicates were prepared, each consisting of primary leaves from three plants.

Fourteen-day-old seedlings from four flasks of each genotype x treatment combination, which were not used for RNA isolation, were carefully taken out of the flasks. The above-ground part was cut, and the root was properly washed from the medium in water. The above-ground parts and roots were scanned in STD4800 Scanner (Regent Instrument). Hypocotyl and primary root length were analyzed in ImageJ (1.53t), while the root length, root surface area, average root diameter, and root volume were measured using WinRhizo Pro (Regent Instruments Inc.). Fresh mass of the above-ground part and the roots was measured in MX analytical scales (Mettler Toledo).

### 2.4 Pot trials in the greenhouse

All stress experiments were conducted in a greenhouse facility (latitude: 45°21′01.0″N, longitude: 11°56′58.6″E, and elevation: 4 m above sea level) situated in the Agripolis Campus of the University of Padua (Italy) during April and May 2024. A total of 100 seeds of each sunflower line (AB-OR-8 and DF-AB-2) were sown in small pots (diameter: 7 cm, height: 6 cm, and volume: 150 mL) and watered regularly until germination. Post-germination, the seedlings with two true leaves were moved to larger pots (diameter: 14 cm, height: 12 cm, and volume: 1,100 mL), with one seedling per pot, with a substrate of peat and river sand in a ratio of 2:1. Plants from both varieties were equally divided into control and stress groups and arranged in the greenhouse following a completely randomized design. Plants were watered regularly until the drought treatment commenced, which occurred when the third and fourth leaves developed.

The drought stress treatment consisted of watering the control group with 300 mL and the stress group with 150 mL every other day, with adjustments made depending on weather conditions. This mild, prolonged stress condition was applied for 10 days (from April 29 to 10 May 2024). During this period, leaf parameters were measured using the DUALEX® optical leaf-clip meter and the LI-600 Porometer on the youngest mature leaves (third and fourth) to monitor plant growth and the physiological responses to water stress. DUALEX® measures chlorophyll, flavonol, and anthocyanin contents, as well as the Nitrogen Balance Index (NBI). The LI-600 is a portable porometer equipped with a Pulse-Amplitude Modulation (PAM) fluorometer that simultaneously measures chlorophyll-a fluorescence and stomatal conductance. By calculating the ratio of chlorophyll to flavonoids, the NBI provides a rapid and accurate estimate of nitrogen availability (de Souza et al., 2022).

At the end of the stress treatment, 30 plants from each variety were randomly selected, which included 15 plants from the control group and 15 from the stress group. These 15 plants were then divided into three groups of five, representing the biological replicates. The third and fourth leaves from each plant were collected and immediately flash-frozen in liquid nitrogen. Leaves belonging to the same replicate were pooled together and stored at −80 °C for RNA extraction and further transcriptomic analysis.

### 2.5 Pot trials in a climate walk-in chamber

A total of 100 seeds of each sunflower inbred line, DF-AB-2 and AB-OR-8, were sown in jiffy pots filled with peat (Klasmann Deilmann TS1). One seed, with the radicle facing downward, was placed in each jiffy pot and transferred to the growth chamber at a constant temperature of 25 °C and in the absence of light. Small 1-dm^3^ pots were filled with the same weight of peat and perlite mixture in a 9:1 ratio, watered to 100% water capacity, and left to drain excess water for 48 h. Three-day-old seedlings from jiffy pots were transferred to the 1-dm^3^ pots, such that one jiffy pot containing one seedling was placed in the middle of one 1-dm^3^ pot. The pots were arranged according to a completely randomized design in a walk-in-chamber with 25 °C, 16-/8-h day/night photoperiod, and 50% relative humidity. In total, 60 pots were placed in the chamber, with 30 pots per genotype. During the first 10 days, pots were watered to maintain near 100% water capacity. On the 10th day, when plants attained the fully developed first pair of leaves, watering of half of the pots of each line was reduced by 50%. Pots with reduced watering were labeled as stress treatment pots and pots continually watered to 100% water capacity as control. Watering was reduced for a period of 10 days. Before sampling plants, stomatal conductance and fluorometric measurements were determined on the youngest fully developed leaves (third and fourth) using an LI-600 Porometer/Fluorimeter (LI-COR Biosciences).

Leaves of the plants were harvested, flash-frozen in liquid nitrogen, and then stored in a deep freeze until RNA isolation. Three biological replicates were prepared for each genotype × treatment combination, each consisting of a bulk sample of three individual plants.

### 2.6 RNA isolation and RNA-seq

All samples for RNA extraction had been flash-frozen in liquid nitrogen immediately after sampling and stored at −80 °C. For each experiment, a different number of replicas were pooled (Supplementary Table 1). The different methods of RNA extraction and their modifications are indicated in Supplementary Table 1. RNA quality and quantity for all samples were checked using a nanophotometer (Nano Photometer® NP80, Implen, Munich, Germany) and by using a 1x TAE agarose gel. RNA-seq for triple biological replications was performed by Novogene GmbH (Munich, Germany) for all samples showing good quality and integrity. Poly-A-enrichment was used for library preparation. Sequencing was performed using Illumina NovaSeq X Plus Sequencing System (Ilumina, San Diego, California, USA) with paired-end reads (PE150). HanXRQr2.0-SUNRISE-2.1 (www.heliagene.org) served as the reference genome, and cleaned reads were mapped using HISAT2 (version 2.2.1) (Mortazavi et al., 2008). As a normalization method for read counts, fragments per kilobase million (FPKM) values were calculated (Trapnell et al., 2010). RSEM (version 1.3.3) was used for the calculation of FPKM values (Li and Dewey, 2011). To analyze the quality of the data and reproducibility of the biological replicates, principal component analysis (PCA) was performed using R version 4.5.1. DEGs were identified using DESeq2 software (version 1.42.0) (Love et al., 2014). False positives were eliminated by calculating the adjusted p-value (padj) with the Benjamini–Hochberg procedure (Benjamini and Hochberg, 1995). To identify significant variations in DEGs between treatments and genotypes, a threshold of padj ≤ 0.05 and |log_2_FoldChange| ≥ 1 was applied.

### 2.7 Identification of genes from ABA synthesis and signaling

Sunflower drought-tolerance candidate genes involved in ABA biosynthesis pathway and signaling were identified using two different approaches: the first included identification of the most significantly expressed genes in the RNA-seq dataset obtained from Shi et al. (2024). The second set of genes involved in the ABA synthesis and signaling pathway was retrieved from Kyoto Encyclopedia of Genes and Genomes (KEGG) (https://www.kegg.jp/) (entry M00372). In case that there were no genes found in the HAN (sunflower) database, genes from tomato (SLY) were retrieved and subjected to blasting against the assembly of the XRQ genome from PacBio data (HanXQR2.0) (Badouin et al., 2017). Protein sequences of the identified DEGs were then retrieved from the HanXQR2.0 assembly database. These sequences were further used to compare them against the HanXQR2.0 protein database to identify similar protein sequences across the sunflower genome (Supplementary Table 2).

### 2.8 Grouping differentially expressed candidate genes

The list of candidate genes was applied to the RNA-seq data from the *in vitro* liquid medium, the *in vitro* solid medium, and the greenhouse pot trials (Supplementary Table 3). The results were sorted into three groups. One group contained all genes that were significant for data sets from the *in vitro* liquid medium and greenhouse pot trials in the comparisons between drought stress and control conditions for both genotypes (|log_2_FoldChange| ≥ 1 and padj ≤ 0.05). These genes represented the general drought stress response that was not related to the genotype or test system. The two other groups specifically compared the two genotypes under both the control and drought stress conditions. The second group consisted of significantly different DEGs between the genotypes, both for the control and stress conditions. This group represents the genes that already show a different expression between the genotypes under control conditions and, thus, may provide the drought-tolerant genotype an advantage in the stress response by showing a different expression even before onset of stress. The third group represents only genes differentially expressed under drought stress conditions between these two genotypes. This group consists of genes that are specifically involved in the different stress responses between the two genotypes and, thus, provides the tolerant genotype an advantage during drought stress (Supplementary Table 4). Genes that showed FPKM values <3 for all replications in one comparison were eliminated.

### 2.9 RT-qPCR/ RNA-seq data validation of DEGs

cDNA synthesis was performed following different protocols (Supplementary Table 1). For qPCR experiments, primers for all candidate genes were designed using the IDT PrimerQuest Tool (Integrated DNA Technologies, Coralville, Iowa, USA). PCR products were less than 150 bp in size, and the annealing temperature was set to 62 °C (Supplementary Table 5). Efficiencies of primers were determined using cDNA dilutions of 1:1, 1:10, 1:100, and 1:1,000 (Supplementary Table 6). RT-qPCRs were performed on different machines using different master mixes (Supplementary Table 1). For each gene, three biological replicates with three technical replications per biological replicate were analyzed. Results were evaluated and visualized using Microsoft Excel (Version 2504) and R (R version 4.5.1). A putative vacuolar fusion protein Mon1 (SAND) (Najafabadi and Amirbakhtiar, 2023, HanXRQr2_Chr14g0656731) and a putative ubiquitinyl hydrolase 1 (HanXRQr2_Chr05g0215411, no significant changes in the expression under all conditions in the RNA-seq data, |log_2_FoldChange| ≤ 0.15) were used as reference genes. They were checked for suitability by comparing qPCR results from different samples. The Pfaffl method was used to calculate the relative gene expression (Pfaffl, 2001).

### 2.10 Statistical analyses

All datasets obtained from the *in vitro* and the pot experiments were tested for normality and homogeneity of variances among groups using the Shapiro–Wilk and Levene’s tests, respectively. As most datasets did not satisfy the assumptions of parametric tests, nonparametric approaches were applied. For physiological parameters and relative gene expression data, group differences were assessed using the Kruskal–Wallis test (Kruskal and Wallis, 1952), which is a rank-based nonparametric alternative to one-way ANOVA that evaluates whether samples originate from the same distribution. When significant effects were detected, pairwise comparisons were performed using the Wilcoxon rank-sum test with Bonferroni correction for multiple testing. All morphological datasets were analyzed using the Games–Howell test (Games and Howell, 1976). A significance level of *p* < 0.05 was applied for all statistical analyses.

Statistical analyses and data visualizations were performed in R version 4.5.1 (R Core Team, 2024) using the packages rstatix, readxl, dplyr, tidyr, purrr, and multcompView. All figures were generated in R using ggplot2 and patchwork, while summary tables were created in Microsoft Excel (Microsoft Office Professional Plus 2016).

## 3 Results

### 3.1 General comparison across the different experimental setups and methods

**FIGURE 1:**
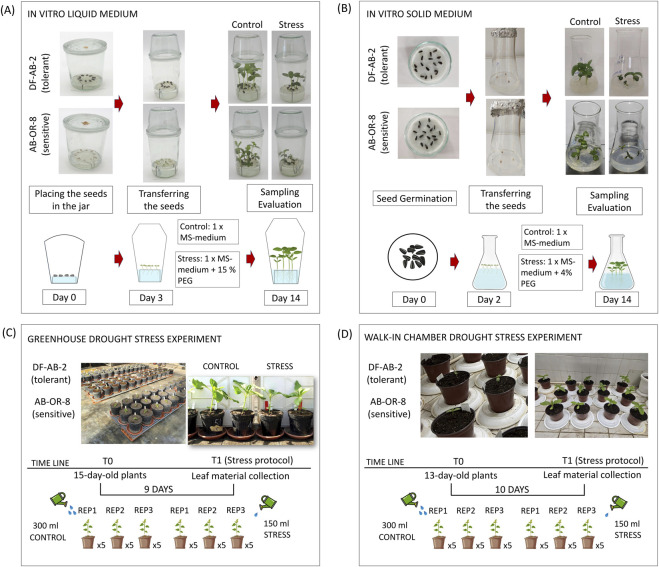
Overview of the four different systems used for testing drought stress in sunflowers. **(A)** *In vitro* system using liquid MS-medium and applying 15% PEG for drought stress conditions. **(B)** *In vitro* system using solid medium and adding 4% PEG as drought stress. **(C)** Soil-based pot trials performed in the greenhouse. **(D)** Soil-based pot trials performed in a walk-in climate chamber.

Two *in vitro* systems were used to simulate drought stress at the seedling stage: PEG-supplemented liquid medium and solid medium (Figure 1). Both allowed controlled application of osmotic stress, but resulted in different stress intensity, root accessibility, and traits that could be reliably measured. In the liquid *in vitro* medium, seeds were placed on a mesh above MS-medium, allowing proper above-ground and root development, with the roots being fully immersed in the medium. This setup ensured consistent PEG exposure (15% PEG) and facilitated easy access to root tissues for morphological analysis. In this way, the liquid system allowed precise quantification of the shoot traits (e.g., hypocotyl length, and leaf area) and detailed root traits (e.g., total root length, surface area, and volume) (Table 1). The solid medium, composed of PEG-infused phytagel (4% PEG), supported vertical seedling growth and allowed straightforward measurement of shoot traits such as hypocotyl length and primary leaf area (Table 1). However, root extraction was more difficult, which is also reflected in the standard deviation of the measured traits compared to the liquid medium. The stress imposed on the plants was mild in the *in vitro* solid medium.

**TABLE 1:** Comparative overview of methods for screening for drought response.

Assessment criteria	*In vitro* liquid medium screening		*In vitro* solid medium screening		Pot trials in the greenhouse		Pot trials in the walk-in climate chamber	
General handling	Skills in sterile working required		Skills in sterile working required		No special skills		No special skills	
Contamination	Sterile work conditions		Sterile work conditions		Not required		Not required	
Sterility of seeds	Necessary		Necessary		Not required		Not required	
Reproducibility	High		High		Moderate		High	
Growing condition	Light-, light cycle-, and temperature-regulated		Light-, light cycle-, and temperature-regulated		Greenhouse conditions between April–May		Light-, light cycle-, and temperature-regulated	
Stress conditions	15% PEG		4% PEG		50% pot field capacity		50% pot field capacity	
Handling of roots for phenotyping	Easy—direct scanning with WinRHIZO™		Difficult—requires washing of the roots		Difficult—requires washing of the roots		Difficult—requires washing of the roots	
Phenotyping shoots—parameters	Fresh weight (shoot, primary leaves, and cotyledons)	g	Fresh weight (shoot, primary leaves, and cotyledons)	g	nd	​	nd	​
	Leaf area (primary leaves and cotyledons)	cm^2^	nd	​	nd	​	nd	​
	Hypocotyl length	cm	Hypocotyl length	mm	nd	​	nd	​
Physiological parameters	nd	​	nd	​	Stomatal conductance	gsw with Licor (mol m^−2^ s^−1^)	Stomatal conductance	gsw with Licor (mol m^−2^ s^−1^)
​	nd	​	nd	​	Apparent transpiration	Licor (mol m^−2^ s^−1^)	Apparent transpiration	Licor (mol m^−2^ s^−1^)
​	nd	​	nd	​	Vapor pressure deficit	KPa	Vapor pressure deficit	KPa
​	nd	​	nd	​	Chlorophyll index	Chlorophyll index Dualex	nd	​
​	nd	​	nd	​	Nitrogen balance index	NBI Dualex	nd	​
Phenotyping roots—parameters	Fresh weight roots	g	Fresh weight roots	g	nd	​	nd	​
	Total root length	cm	Total root length	cm	nd	​	nd	​
	Primary root length	nd	Primary root length	cm	nd	​	nd	​
	Surface area	cm^2^	Surface area	cm^2^	nd	​	nd	​
	Root volume	cm^3^	Root volume	cm^3^	nd	​	nd	​
	Average diameter	mm	Average diameter	mm	nd	​	nd	​
Costs	Moderate		Moderate	​	Low		Low	
Duration	3 days of germination and 11 days of stress treatment		2 days of germination and 12 days of stress treatment	​	14 days of germination and seedling growth and 10 days of stress treatment		14 days of germination and seedling growth and 10 days of stress treatment	
Sampling for biochemical analyses	Both for shoots and roots		Shoots	​	Shoots		Shoots	
RNA isolation	Fast, easy sampling for shoots and roots, stored at −80 °C		Fast, easy sampling only for shoots, stored at −80 °C	​	Fast, easy sampling only for shoots, stored at −80 °C		Fast, easy sampling only for shoots, stored at −80 °C	
RNA-seq	Both for shoots and roots		Shoots	​	Shoots		Shoots	
Requirements	Climate chamber or cabinet		Climate chamber or cabinet	​	Greenhouse		Walk-in climate chambers	

nd, not determined.

The two pot-based systems, greenhouse and walk-in climate chamber, enabled drought stress simulation under soil-grown conditions, allowing additional measurement of physiological parameters (Table 1). Stress was applied by controlled water retention, but the environmental conditions differed substantially between the two setups (Figure 1). In the greenhouse, plants were exposed to natural fluctuations in temperature, humidity, and light. These conditions intensified the drought stress, leading to more profound physiological responses. This setup imposed a stronger stress signal, which became particularly evident in the susceptible genotype AB-OR-8. The climate chamber provided tightly controlled environmental conditions, with stable temperature, humidity, and light. This resulted in more gradual and reproducible stress responses. The imposed stress was milder in the climate chamber than in the greenhouse.

### 3.2 Differences in the morphological traits across the two *in vitro* systems

**FIGURE 2:**
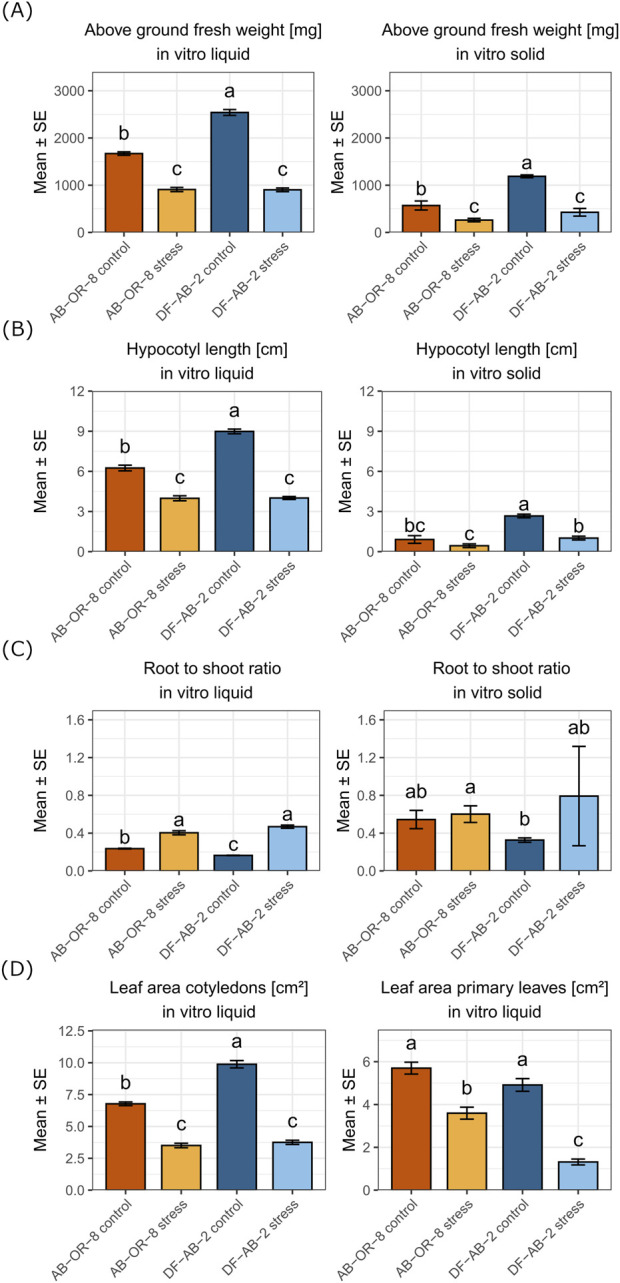
Comparison of shoot parameters from liquid and solid *in vitro* systems of 14-day-old sunflower seedlings. AB-OR-8 control conditions (dark orange), AB-OR-8 drought stress (light orange), DF-AB-2 control conditions (dark blue), and DF-AB-2 drought stress (light blue). **(A)** Above-ground fresh weight [mg]; **(B)** hypocotyl length [cm]; **(C)** root-to-shoot ratio; **(D)** leaf area of cotyledons and primary leaves [cm^2^]. The data are represented as the mean ± standard error of three independent biological replicates. Statistical significance (*p* < 0.05) was evaluated by the Games–Howell test.

Comparing shoot traits, both *in vitro* systems showed similar results (Figure 2). In both the genotypes, above-ground fresh weight was higher under control conditions than under drought conditions, while the tolerant genotype DF-AB-2 already started with a higher above-ground fresh weight under control conditions. Under drought stress, both genotypes showed no significant differences. In general, the plants in the *in vitro* system with solid MS-medium weighed lesser and yielded shorter hypocotyls than the ones from the *in vitro* system with liquid MS-medium. The same trend was observed for the hypocotyl length. DF-AB-2 had larger cotyledons but smaller primary leaves than AB-OR-8, which were only measured in the *in vitro* liquid system.

The root-to-shoot ratio only showed significant differences between the control and drought conditions in the liquid *in vitro* system, but not in the solid *in vitro* system (Figure 2). Both genotypes had higher root-to-shoot ratios under drought stress conditions, due to a decrease in shoot biomass compared to root biomass. This shift in biomass was more pronounced in the tolerant genotype DF-AB-2 as it had a lower root-to-shoot ratio under control conditions than for the drought-sensitive genotype AB-OR-8.

**FIGURE 3:**
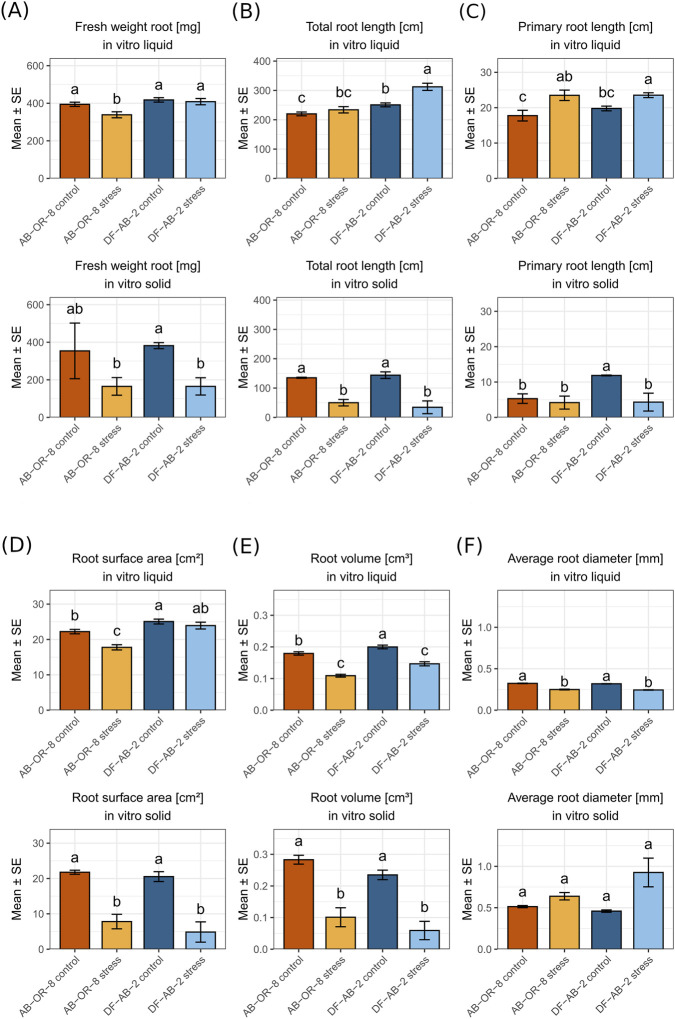
Comparison of root parameters from liquid and solid *in vitro* systems of 14-day-old sunflower seedlings. AB-OR-8 control conditions (dark orange), AB-OR-8 drought stress (light orange), DF-AB-2 control conditions (dark blue), and DF-AB-2 drought stress (light blue). **(A)** Fresh weight root [mg]; **(B)** total root length [cm]; **(C)** primary root length [cm]; **(D)** root surface area [cm^2^]; **(E)** root volume [cm^3^]; **(F)** average root diameter [mm]. The data are represented as mean ± standard error of three independent biological replicates. Statistical significance (*p* < 0.05) was evaluated by the Games–Howell test.

Comparing the root traits, AB-OR-8 and DF-AB-2 showed different responses to the drought stress applied by the two *in vitro* systems (Figure 3). In the liquid *in vitro* system, the main drought response observed for the root system was a considerably increased total root length in the tolerant genotype DF-AB-2 and longer primary root length for both genotypes under drought stress. In the solid *in vitro* system, a reduction in all measured root traits under drought stress, except for the average diameter, was observed for both genotypes. Comparing the two *in vitro* drought-screening systems, the roots from the solid *in vitro* system were shorter, but roots from both systems showed similar weight in the controls.

### 3.3 Differences in the physiological parameters

**FIGURE 4:**
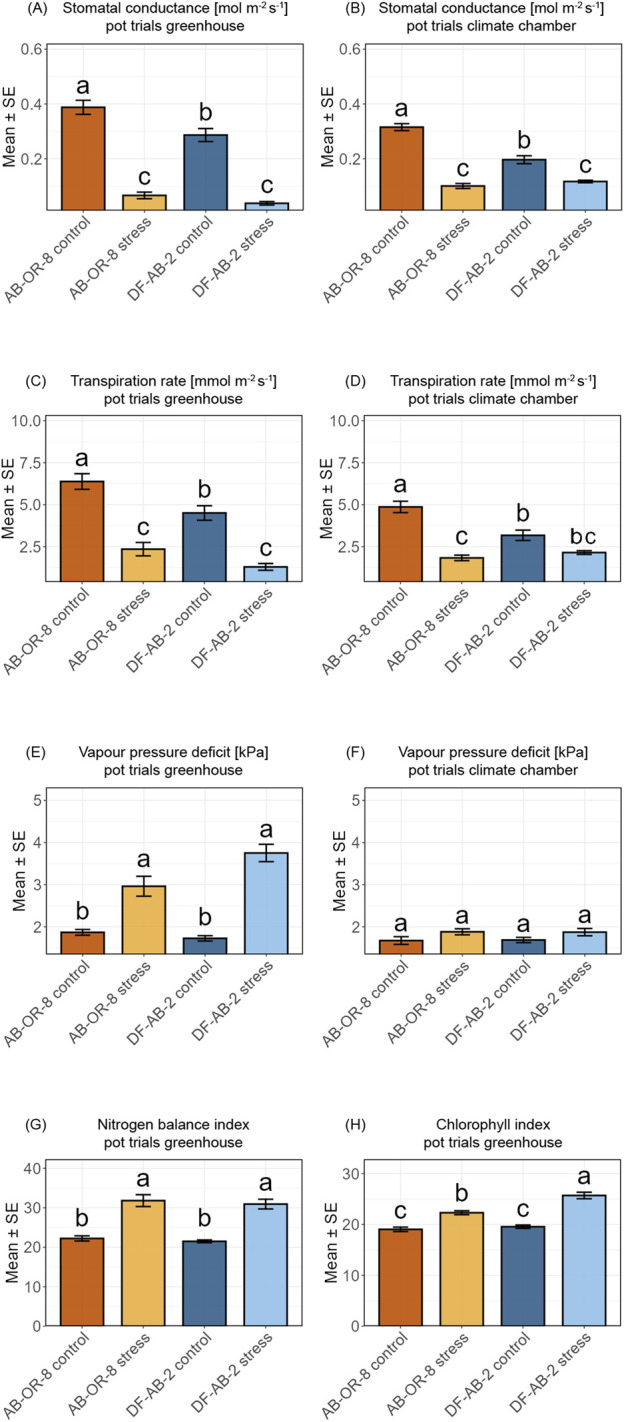
Comparison of physiological parameters from pot trials for drought stress performed in the greenhouse (GH) or in a walk-in climate chamber (CC). AB-OR-8 control conditions (dark orange), AB-OR-8 drought stress (light orange), DF-AB-2 control conditions (dark blue), and DF-AB-2 drought stress (light blue). The data are represented as the mean ± standard error of three independent biological replicates. Statistical significance (*p* < 0.05) was evaluated by the Kruskal–Wallis test followed by a Wilcoxon *post hoc* test with Bonferroni correction. **(A)** Stomatal conductance [mol m^−2^ s^−1^] GH. **(B)** Stomatal conductance [mol m^−2^ s^−1^] CC. **(C)** Transpiration rate rate [mmol m^−2^ s^−1^] GH. **(D)** Transpiration rate [mmol m^−2^ s^−1^] CC. **(E)** Vapour pressure deficit [kPa] GH. **(F)** Vapour pressure deficit [kPa] CC. **(G)** Nitrogen balance index GH. **(H)** Chlorophyll index GH.

On comparing physiological traits, both the pot systems showed clear responses to drought stress, but there were differences in magnitude and consistency (Figure 4). In both environments, stomatal conductance decreased under drought stress in both genotypes, with the sensitive genotype AB-OR-8 showing the lowest values. In the greenhouse, this reduction was more pronounced, especially in AB-OR-8 (Figure 4A). In the climate chamber, the same trend was observed, but the differences between the genotypes were less distinct (Figure 4B).

The transpiration rate followed a similar pattern. In the greenhouse, drought stress led to a significant reduction in transpiration in both genotypes, with AB-OR-8 again showing the strongest response (Figure 4C). In the climate chamber, the reduction was present only in AB-OR-8, while DF-AB-2 showed no significant difference between the control and drought stress conditions (Figure 4D).

Vapor pressure deficit increased under drought stress in the greenhouse for both genotypes, with AB-OR-8 showing the highest values under stress (Figure 4E). In the climate chamber, no significant differences were observed between the treatments or genotypes (Figure 4F), indicating a more stable vapor environment and milder stress intensity.

The nitrogen balance index and chlorophyll index were only measured in the greenhouse. Here, drought stress led to a significant increase in both traits in both genotypes. The increase in nitrogen balance index under drought was almost identical between the genotypes, with both showing similar values under control conditions and similar values under drought (Figure 4G), whereas for the chlorophyll index, this increase was more pronounced in the tolerant genotype DF-AB-2 (Figure 4H). This indicates that DF-AB-2 maintained or enhanced the pigment levels more strongly under stress, which may reflect a better capacity to sustain photosynthetic activity compared to AB-OR-8.

### 3.4 Genes of the ABA signaling pathway as indicators for drought stress

A list of 109 genes consisting of 46 genes involved in ABA biosynthesis and 63 genes in the ABA signaling pathway was compiled to identify candidate genes for drought response by applying information from Shi et al. (2024) and additional information from KEGG (Supplementary Table 2).

**FIGURE 5:**
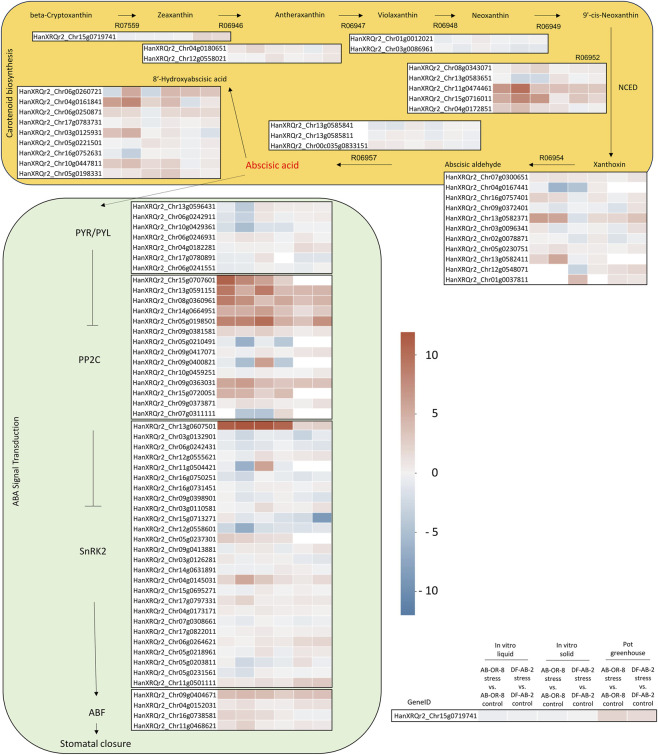
Differential gene expression of genes belonging to the ABA biosynthesis and signaling in two sunflower genotypes, AB-OR-8 and DF-AB-2, comparing the control and drought conditions.

RNA-seq data were obtained for the two *in vitro* systems and the soil-based pot trials in the greenhouse (Figure 5; Supplementary Table 3). Expressions of genes encoding 9-cis-epoxycarotenoid dioxygenases (NCEDs) that catalyze the rate-limiting step converting 9′-cis-neoxanthin to xanthoxin are the most relevant in ABA biosynthesis. Especially, in the *in vitro* liquid system, the drought-tolerant DF-AB-2 exhibited a stronger upregulation of the expressions levels of three NCED encoding genes, namely, HanXRQr2_Chr11g0474461, HanXRQr2_Chr15g0716011, and HanXRQr2_Chr4g0172851, under water deficit than the sensitive genotype AB-OR-8. The expression levels of these genes were also upregulated under drought for both genotypes in the pot trials in the greenhouse, but to a lesser degree. As part of ABA biosynthesis, upregulation might indicate a faster production of ABA as a reaction to water deficit, especially by the drought-tolerant line DF-AB-2.

With regard to the ABA signaling pathway, three genes (HanXRQr2_Chr13g0596431, HanXRQr2_Chr06g0242911, and HanXRQr2_Chr10g0429361) that encode for the ABA receptors PYR/PYL were more downregulated more in DF-AB-2 under drought conditions in the *in vitro* liquid system than in AB-OR-8.

The PP2C gene family represents a large gene family with 132 members in sunflower (Supplementary Figure 1). Our study presents the results for 16 PP2C members that belong to clade A, which had been described in *Triticum aestivum* to contain PP2Cs reacting to abiotic stress (Supplementary Figure 2; Yu et al., 2019). Seven of these PP2C genes were differentially upregulated in all three analyzed screening systems. Notably, AB-OR-8 showed a stronger upregulation than DF-AB-2 for at least three of these genes (HanXRQr2_Chr15g0707601, HanXRQr2_Chr13g0591151, and HanXRQr2_Chr8g0360961) in the *in vitro* liquid system. Members of the SnRK2 gene family (26 members) showed very strong upregulation for HanXRQr2_Chr13g0607501 for both sunflower lines in both *in vitro* systems accompanied with a weaker reaction in the pot trials in the greenhouse. In general, the regulation of the ABA biosynthesis and signaling pathway was more pronounced in both *in vitro* systems than in the soil-based pot trials conducted in the greenhouse.

For the selection of genes that might best represent the stress level applied by the four drought stress selection systems, five different comparisons were performed (Supplementary Table 4): AB-OR-8 stress vs. AB-OR-8 control from the liquid *in vitro* system, DF-AB-2 stress vs. DF-AB-2 control for both the liquid *in vitro* systems and the pot trials from the greenhouse, both genotypes under control conditions, and both genotypes under drought stress conditions for the liquid *in vitro* system. A total of 14 genes that showed significant differences in any of these comparisons were selected for verification by quantitative RT-PCR (Supplementary Table 5). Six genes demonstrating the best primer efficiencies were finally chosen for further experiments (Supplementary Table 6).

**FIGURE 6:**
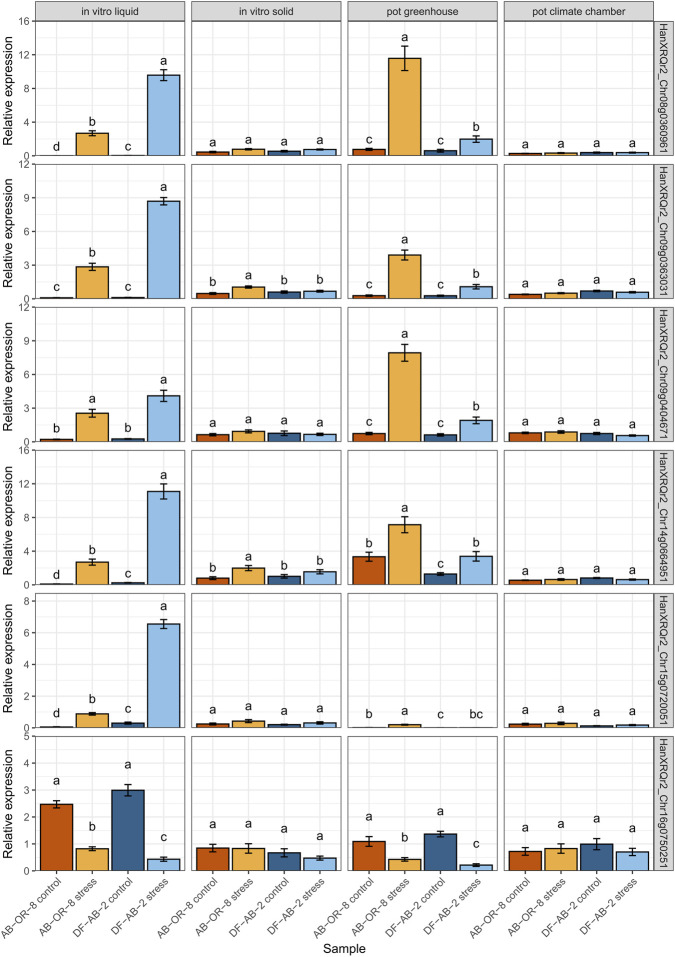
Comparison of relative gene expressions for six genes of ABA signaling in the four different screening systems used for drought stress in sunflower. AB-OR-8 control conditions (dark orange), AB-OR-8 drought stress (light orange), DF-AB-2 control conditions (dark blue), and DF-AB-2 drought stress (light blue). The data are represented as mean ± standard error of three independent biological replicates, with three technical replications per biological replication. Statistical significance (*p* < 0.05) was evaluated by the Kruskal–Wallis test followed by a Wilcoxon *post hoc* test with Bonferroni correction.

These selected genes of the ABA signaling pathway were then used to compare the four drought-simulating systems in order to analyze the level of drought stress imposed by each system (Figure 6), respectively. RT-qPCRs were performed for all six genes (HanXRQr2_Chr08g0360961, HanXRQr2_Chr09g0363031, HanXRQr2_Chr09g0404671, HanXRQr2_Chr14g0664951, HanXRQr2_Chr15g0720051, and HanXRQr2_Chr16g0750251) in both lines, AB-OR-8 and DF-AB-2. Five of the genes were upregulated under drought conditions in two of the systems (*in vitro* liquid medium and pot trials in the greenhouse). Four of these genes encode for PP2Cs (HanXRQr2_Chr08g0360961, HanXRQr2_Chr09g0363031, HanXRQr2_Chr14g0664951, and HanXRQr2_Chr15g0720051), and one encodes for a bZIP transcription factor (HanXRQr2_Chr09g0404671). In the *in vitro* solid system, but not in the pot trials in the climate chamber, HanXRQr2_Chr09g0363031 and HanXRQr2_14g0664951 were upregulated for the drought-sensitive genotype, but not for the drought-tolerant genotype. Downregulation of HanXRQr2_Chr16g0750251 was also only observed in the *in vitro* liquid medium and pot trials in the greenhouse under drought conditions. This gene encodes a CAMK-OSTIL. The differential RT-qPCR results demonstrate that the *in vitro* liquid systems imposed the strongest drought stress, followed by the pot trials in the greenhouse. The *in vitro* solid system only induced very mild stress, which led to an upregulation for the drought-sensitive AB-OR-8 but not for DF-AB-2 in two genes. The drought stress in the pot trials in the climate chamber was so mild that no drought response could be detected by RT-qPCR of the six genes.

## 4 Discussion

Breeding for drought-tolerance requires efficient screening methods that allow analyzing large populations or a large number of genotypes in a high-throughput fashion. Using PEG for drought simulation represents an interesting alternative to field trials as it requires considerably less space along with controlled growing conditions. However, applying PEG for drought-screening raises three major critical questions: (1) What concentration of PEG should be used to induce drought stress? (2) How should PEG be applied? (3) How comparable are the results between PEG and field trials for predicting drought tolerance? Few publications compare different methods for screening for drought response in general, with most being reviews contrasting various experimental setups with different genotypes or even species (Osmolovskaya et al., 2018; Marchin et al., 2020; Wang et al., 2024).

*In vitro* systems using PEG to simulate drought stress are widely applied in early-stage screening of drought tolerance due to their high reproducibility and precise control over water availability (Huynh et al., 2014; Wu et al., 2017). Between 5% and 20% of PEG can be applied to reduce the water potential. Habtegebriel et al. (2025) tested 0% (control), 5% (mild drought stress), 10% (moderate drought stress), and 20% (severe drought stress) PEG in barley. Significant effects of genotype and drought stress levels were observed for most of the studied traits. The highest percentage of 20% PEG was also used for drought screening in another study in barley (Cai et al., 2020), in *Vicia sativa* (Gonzalez et al., 2025) and for investigating the drought response in sunflower (Fulda et al., 2011). However, in tomato, PEG concentrations between 2% and 12% were tested, and 4% was regarded as optimal for screening (George et al., 2015). Applying the optimal drought stress is crucial for the efficiency of drought screening.

A variety of options exists on how to apply PEG: (1) PEG can be combined with a solid medium; (2) it can be used to soak paper towels (George et al., 2015; Hasanuzzaman et al., 2017); (3) vermiculite can be washed with PEG (Cai et al., 2020); and (4) PEG can be used in a liquid medium. PEG in a liquid medium can be very viscous, thus hampering the supply of oxygen and nutrients to the roots (Munns et al., 2010). Using vermiculite washed with PEG circumvents this problem of viscosity and its consequences (Cai et al., 2020).

The two protocols used in our study not only differed in the amount of PEG applied (15% in the liquid medium and 4% in the solid medium) but also differed in the way PEG was added to the MS-medium. PEG was either autoclaved with the liquid MS-medium or filter-sterilized and then added to the sterile MS-medium before hardening. The process of autoclaving PEG is known to cause chemical degradation and increase the acetaldehyde content, thus potentially intensifying osmotic stress (Kavanagh et al., 1990). Sarmadi et al. (2019) emphasized that PEG’s impact on plant tissue cultures is highly sensitive to its concentration and handling, reinforcing the importance of the sterilization method in experimental design.

Assessing the relevance of PEG-based drought-screening for field trials, Cai et al. (2020) compared both water deficit and PEG-simulated drought in vermiculite as the support in barley. A high correlation between the two systems was observed regarding the reduction of shoot fresh weight, dry weight, and water content for all 427 barley genotypes (cultivated and wild). In addition, 18 contrasting barley genotypes selected by the two systems were assessed with regard to biomass production under water-deficit conditions. All investigated traits (above-mentioned traits and measurements of photosynthetic activity and osmotic adjustment) were significantly correlated with biomass and represented reliable selection parameters for drought tolerance (Cai et al., 2020). Gonzalez et al. (2025) combined *in vitro* screening tests with 20% PEG with field trials in *Vicia sativa*. The root and shoot dry weights of the *in vitro* experiments were positively correlated with yield traits in the field, which makes them valuable early predictors of agronomic performance. Hasanuzzaman et al. (2017) performed different drought treatments in the greenhouse and measured photosynthesis activities, transpiration, dry biomass, shoot water content, and relative root and shoot lengths. A strong correlation was observed between the chlorophyll fluorescence Fv/Fm ratio and drought tolerance. However, the relative root length in the genotypes screened with 18% PEG as paper-roll-grown seedlings appeared to be a reliable and easy method to measure the parameter for breeding programs as well (Hasanuzzaman et al., 2017).

To our knowledge, our study is the first that evaluates several drought-screening setups across three laboratories using identical genotypes and thereby provides a unique opportunity to compare system performance across morphological, physiological, and molecular traits in the same material, with regard to sunflower.

Shoot and root morphological traits were compared in sunflower seedlings grown under drought stress in two *in vitro* systems: liquid MS-medium with 15% PEG and solid MS-medium with 4% PEG. Shoot traits such as above-ground fresh weight, hypocotyl length, and leaf area were consistently higher in the liquid medium under both control and stress conditions. Although the PEG concentration was lower in the solid medium (4%) than in the liquid medium (15%), plants exhibited more reduced shoot and root growth under stress. In the liquid medium, plants exhibited longer primary roots, increased total root length, and higher root volume and surface area, suggesting enhanced elongation and acquisition of water and nutrition under drought conditions. These differences align with earlier findings indicating that liquid media generally promote stronger shoot and root development in plant species (Mbiyu et al., 2011; Rezali et al., 2017). Moreover, solid media tend to intensify the impact of PEG on growth parameters (Ebrahim et al., 2006). In our study, the average root diameter was lesser in the liquid medium, indicating thinner roots (Figure 3). In the solid *in vitro* medium, root systems were more compact and constrained, with reduced volume and surface area. This may be explained by the solidified media imposing mechanical impedance that hinders root penetration and lateral spread, thereby limiting overall biomass accumulation (George et al., 2007; Zheleznichenko et al., 2023). In contrast to the solid medium, the liquid medium allows unrestricted root growth and more uniform stress exposure, which, despite the higher PEG concentration, may support root elongation and shoot development. This demonstrates that growth outcomes are shaped not only by PEG concentration but also by the medium structure and PEG preparation protocols. In our study, the liquid *in vitro* system using 15% PEG imposed the strongest osmotic stress and was the easiest to handle, especially regarding root traits and molecular studies, making this system well-suited for high-throughput screening. In addition, the drought-tolerant DF-AB-2 developed significantly longer primary roots under drought stress than drought-sensitive AB-OR-8, indicating that the primary root length can be chosen for drought tolerance using the liquid *in vitro* system. The solid *in vitro* system (4% PEG) imposed a very mild stress, but worked well to evaluate shoot traits.

The two soil-based pot trials offered advantages for measuring physiological traits such as stomatal conductance, apparent transpiration, chlorophyll index, vapor pressure, and nitrogen balance index, which could not be measured using the *in vitro* systems. These traits are valuable indicators for drought stress and provide additional information about the physiological conditions of the plants. The greenhouse imposed the strongest soil-based stress regarding both the physiological and the molecular responses. However, the pot trials in the climate chamber with better controlled conditions produced more reliable physiological results compared to the greenhouse. Due to the low drought stress imposed in the climate chamber, only a weak response of the ABA genes was observed, and stronger stress application is required in future studies.

Physiological traits such as gas exchange, vapor pressure deficit and nitrogen allocation play a central role in plant drought acclimation. In this study, these parameters were evaluated in drought-tolerant and susceptible genotypes grown in pots under drought stress in two distinct controlled environments: a semi-controlled greenhouse and a fully controlled walk-in growth chamber. This dual setup allowed assessment of genotype-specific responses under varying degrees of environmental control while the majority of other parameters were kept as similar as possible between the setups, including pot size, the timing of drought initiation, and the protocol used to maintain drought conditions. Gas exchange parameters, including stomatal conductance and transpiration rate, declined significantly under drought conditions in both genotypes and environments, reflecting a typical regulatory response to water deficit. AB-OR-8 exhibited higher stomatal conductance under control conditions, along with more pronounced reduction under stress, suggesting a less conservative strategy of water use. DF-AB-2 maintained a lower baseline in stomatal conductance and showed minimal changes in transpiration in the walk-in chamber, indicating a tighter stomatal control. These patterns align with previous findings that drought-tolerant sunflower genotypes often exhibit reduced stomatal aperture and transpiration to conserve water (Chen et al., 2023; Ashraf and Siddiqi, 2024).

Vapor pressure deficit increased significantly under drought in the greenhouse but remained stable in the walk-in chamber. This contrast likely reflects the degree of environmental control: the greenhouse, subject to natural fluctuations, may have experienced higher temperatures and lower relative humidity, amplifying evaporation and intensifying drought stress. In contrast, the walk-in chamber maintained stable temperature, humidity, and light conditions, which likely buffered the severity of drought. This interpretation aligns with those of recent studies showing that less controlled environments can enforce water-deficit symptoms and complicate genotype comparisons (Moshelion et al., 2024; Ali et al., 2025). Furthermore, Massonnet et al. (2010) reported that even moderate variations in growing conditions, including temperature, light quality, and plant handling, can induce significant differences in phenotypes and molecular profiles in Arabidopsis. The nitrogen balance index and chlorophyll index, measured only in the greenhouse, increased significantly under drought in both genotypes, with DF-AB-2 showing a more pronounced elevation. Our study, suggests an adaptive nitrogen allocation strategy, which potentially enhances photosynthetic resilience under stress. Nitrogen remobilization toward chlorophyll synthesis is a known drought-acclimation mechanism, supporting carbon assimilation for reduced stomatal conductance (Ashraf and Siddiqi, 2024; Ronchi et al., 2025).

ABA biosynthesis and signaling play an important role in the response of plants to drought stress (Aslam et al., 2022). This study demonstrates how both the genotype and experimental setup markedly modulate ABA pathway gene expression under drought stress in sunflower. Differential responses observed between the drought-sensitive AB-OR-8 and drought-tolerant DF-AB-2 genotypes across *in vitro* liquid and solid media, along with the greenhouse and walk-in chamber pot experiments, provide important insights into the flexibility and specificity of ABA signaling as a drought-response mechanism. Gene expressions of key ABA signaling pathway components showed pronounced variation not only between genotypes but also between experimental conditions. The *in vitro* liquid MS-medium supplied with a higher PEG concentration caused pronounced upregulation of some of the ABA signaling genes, especially in the drought-tolerant DF-AB-2 under stress. However, a moderate induction was observed in solid MS-medium. The magnitude of upregulation was genotype-dependent, with DF-AB-2 generally demonstrating a stronger and more consistent ABA response, corresponding with its higher drought tolerance. The difference between the genotypes and the difference in the intensity of the upregulation might be explained by the difference in the intensity of the applied stress. In this case, the liquid MS-medium produced the highest stress, resulting in strong upregulation, especially in the drought-tolerant genotype DF-AB-2. The solid MS-medium and the pot trials in the greenhouse produced milder stress that resulted in a less pronounced upregulation of genes, where the sensitive genotype often showed a stronger response.

The rate-limiting step of the ABA biosynthesis is catalyzed by NCED (Thompson et al., 2000). So the higher expression of the three genes (HanXRQr2_Chr11g0474461, HanXRQr2_Chr15g0716011, and HanXRQr2_Chr4g0172851) by the drought-tolerant DF-AB-2 indicates the ability to produce more ABA, which is important for drought tolerance. These findings are in line with those of recent transcriptomic studies highlighting that effective activation of ABA pathways is correlated with enhanced drought resilience in sunflower and other crops (Liang et al., 2017; Shi et al., 2024).

Interestingly, genes corresponding to gene families like in the case of PP2C displayed a differential behavior of gene members. In sunflower, the PP2C gene family consists of 132 members, of which 16 belong to clade A. Yu et al. (2019) showed that in *Triticum aestivum,* the PP2Cs of clade A are involved in abiotic stress responses. In wheat, eight of the genes in clade A were upregulated after treatment with ABA, and in yeast, two-hybrid assays showed some interactions with SnRK2 members (Yu et al., 2019). Shi et al. (2024) performed weighted gene co-expression network analyses (WGCNA) and identified five PP2C genes that were upregulated under drought stress and belonged to clade A. PP2C genes are known to be differentially expressed under drought stress (Yu et al., 2019). For short stress periods, PP2C is downregulated to promote the activation of SnRK2 and leads to stomatal closure. Later, PP2C is upregulated to form a feedback loop to work against an overstimulation of ABA signals. Yang et al. (2018) found significantly upregulated genes of the PP2C family in *Medicago truncatula* within a few hours after drought and ABA treatment.

One gene (HanXRQr2_Chr16g0750251), which was downregulated, encodes for a CAMK-OST1L. This gene was also described as being downregulated under water deficit conditions in barley (*Hordeum vulgare* L.) (Harb et al., 2020). This is consistent with the results of our study throughout all the screening systems.

For comparison of the four different drought-screening systems, using the same two sunflower genotypes, we tested several genes involved in ABA-mediated drought response to validate the drought stress levels induced by the four different screening systems. Regarding the six selected genes of the ABA signaling pathway (HanXRQr2_Chr08g0360961; HanXRQr2_Chr09g0363031; HanXRQr2_Chr09g0404671; HanXRQr2_Chr14g0664951; HanXRQr2_Chr15g0720051, and HanXRQr2_Chr16g0750251), using quantitative RT-PCR, the stress level, which each system screening for drought-tolerance represented, could be determined. The liquid *in vitro* system and the pot trials from the greenhouse indicated similar stress levels, whereas the solid *in vitro* system and the pot trials in the walk-in climate chamber showed weaker stress levels, in which only the drought-sensitive genotype, if at all, demonstrated a differential higher expression. The drought-tolerant genotype did not signal a drought response in these two systems. Even though the *in vitro* systems and the pot trials were set up to represent the same drought selection, it became clear that the liquid and the solid *in vitro* systems differed in the stress applied. The solid *in vitro* medium generated too mild stress conditions. For the pot trials, the plants in the greenhouse experienced more variation regarding temperature and light conditions compared to the walk-in chambers, which resulted in less drought stress in the latter system. In the selection for drought tolerance, it is very important to apply the correct stress level, which allows a differentiation between drought-sensitive and drought-tolerant genotypes. Shi et al. (2024) had also included the ABA biosynthesis pathway and signaling in RNA-seq analyses and had also identified four of the genes in our study (HanXRQr2_Chr08g0360961, HanXRQr2_Chr09g0363031, HanXRQr2_Chr14g0664951, and HanXRQr2_Chr15g0720051). However, two additional genes, one upregulated (HanXRQr2_Chr09g0404671) and one downregulated (HanXRQr2_Chr16g0750251), were identified as new interesting stress indicators for all systems in our study. Comparing our RNA-seq results with Shi et al. (2024), we obtained much stronger upregulation (log_2_FoldChanges above 10), while the differences between the genotypes were not as pronounced and consistent over the treatments. Shi et al. (2024) detected strong differences between the analyzed genotypes. The observed differences in expression change and intensity may be because of the difference in stress duration and application. While the stress applied by Shi et al. (2024) lasted for only 72 h, the stress applied in our study lasted for nine to 12 days.

Pot trials conducted in the greenhouse and walk-in climate chamber environments revealed that drought-induced ABA gene expression was generally more pronounced in greenhouse-grown samples than in the highly regulated chamber setting. This is likely attributed to the greater environmental variability of the greenhouse (e.g., higher temperature and vapor pressure deficit fluctuations), which can enhance the perceived stress and, thus, the activation of the ABA pathway (Moshelion et al., 2024; Prosper et al., 2024). In the walk-in chamber, more stable conditions were associated with a reduced ABA-mediated stress response, further highlighting how the experimental context shapes physiological and molecular outcomes. Moreover, it contributes to the results obtained from measuring the physiological parameters, suggesting that the drought stress in the walk-in chamber was too mild.

Consistent upregulation of ABA signaling genes in drought-tolerant DF-AB-2, especially under *in vitro* liquid medium and greenhouse drought treatments, substantiates that a robust ABA pathway activation is a hallmark of drought-tolerant sunflower genotypes. This is reflected in both classical and recent studies associating elevated expression of ABA-related genes (NCED, PP2C, SnRK2s, DREB, etc.) with enhanced drought acclimation in sunflower, along with downstream physiological effects such as reduced stomatal conductance and improved water-use efficiency (Rauf, 2008; Ashraf and Siddiqi, 2024; Shi et al., 2024; Wang et al., 2024).

In this paper, we have described four well-established systems for drought stress screening in sunflower that allow reliable measurement of multiple morphological and physiological traits under controlled drought conditions. By standardizing drought exposure and trait quantification, these systems create a robust foundation for downstream allele mining aimed at identifying genomic variants associated with improved drought tolerance in sunflower. To fully exploit this potential, future work will require evaluation of a substantially larger and more genetically diverse association panel, as demonstrated in other crops, where expanded GWAS populations have significantly increased the ability to detect adaptive alleles (Radanović and Horn, 2024).

Recent integrative studies combining GWAS with transcriptomic profiling have already highlighted the complexity of drought-response pathways in sunflower and demonstrated the feasibility of identifying candidate genes and regulatory modules underlying stress adaptation (Wu et al., 2022a). In particular, ABA-signaling components, including abscisic acid-related protein kinases and transcription factors, have emerged as key regulators of drought responses, and expanding association mapping to broader germplasm sets should enable more precise dissection of their functional variations.

The drought-screening systems described here, which allow high-throughput phenotyping coupled with large-scale genomic resources and integrative analytical approaches, provide strong platforms for accelerating the discovery of drought-tolerance alleles in sunflower. Within this framework, the ABA signaling genes identified in our study represent particularly promising entry points for allele mining. Key components of ABA biosynthesis and signaling (such as NCEDs, PYR/PYL receptors, PP2Cs, and SnRK2s) are well-established regulators of stomatal control, hydraulic adjustment, and osmotic protection during drought, and natural variation in these pathways has been linked to differential stress tolerance in multiple crops (Cutler et al., 2010; Chen et al., 2020; Lim et al., 2022; Mo et al., 2024; Wei et al., 2025). Associating the allelic variants in these genes with the multi-trait phenotypic data generated by our four screening systems could clarify genotype-specific drought-response strategies and uncover additional, currently unknown regulatory or structural loci involved in acclimation. Such insights would provide breeders with validated molecular targets and diagnostic SNPs that are suitable for integration into marker-assisted and genomic-selection pipelines, ultimately supporting the development of more drought-resilient sunflower cultivars.

## Data Availability

NGS data generated for this study are stored under BioProject ID PRJNA1354464, BioProject ID PRJNA1369986, and BioProject ID PRJNA1373301 in the NCBI Sequence Read Archive (https://www.ncbi.nlm.nih.gov/sra/).
