# Supplementary figures and images for: Toward efficient screening systems for sunflower drought tolerance: a comparative and molecular perspective
Source: Front Bioeng Biotechnol. 2026 Feb 19;13:1735259. doi: 10.3389/fbioe.2025.1735259 (PMC12961277; doi:10.3389/fbioe.2025.1735259)

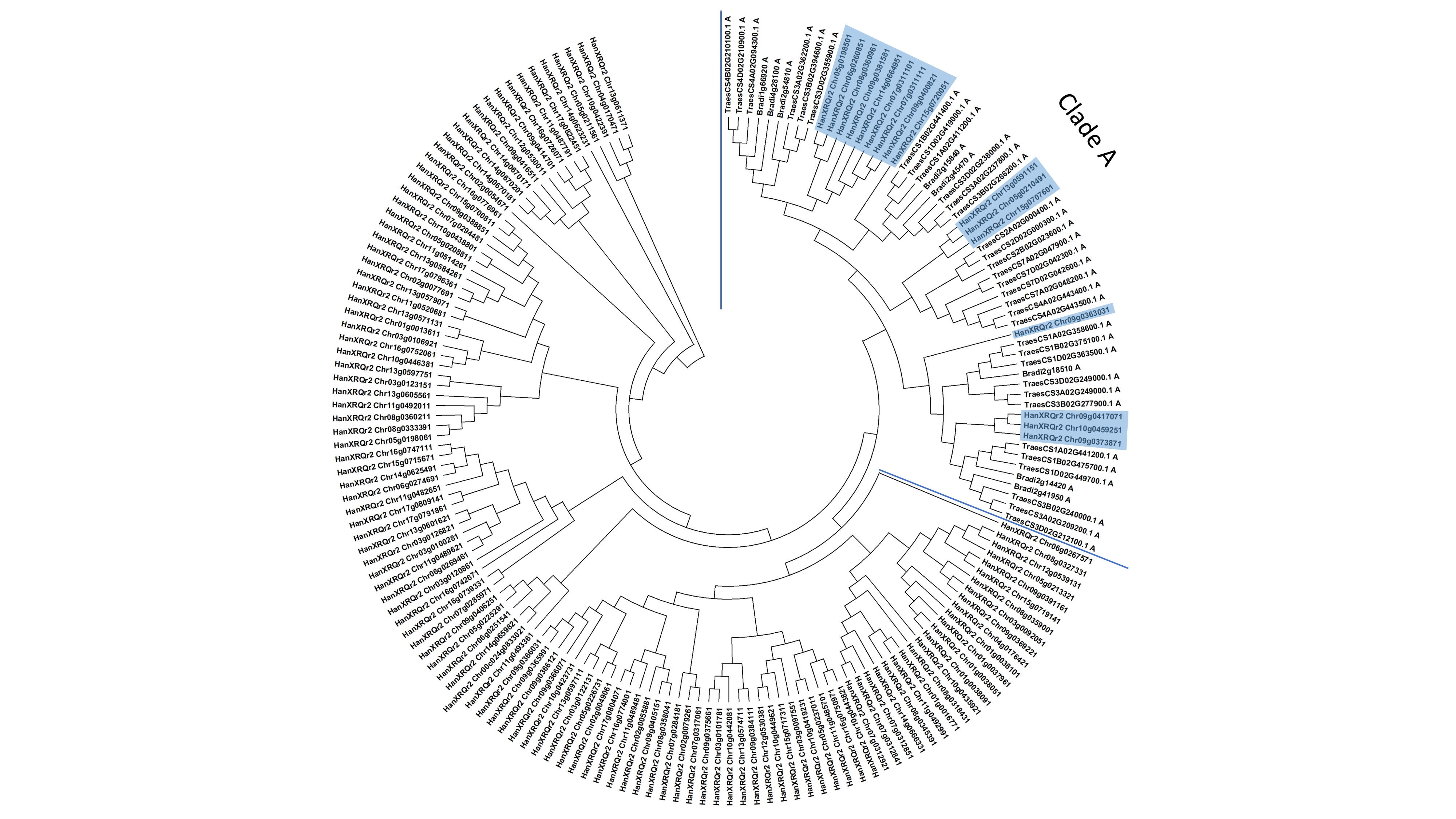

Supplement: Supplementary file 3 [file Image2.tif]

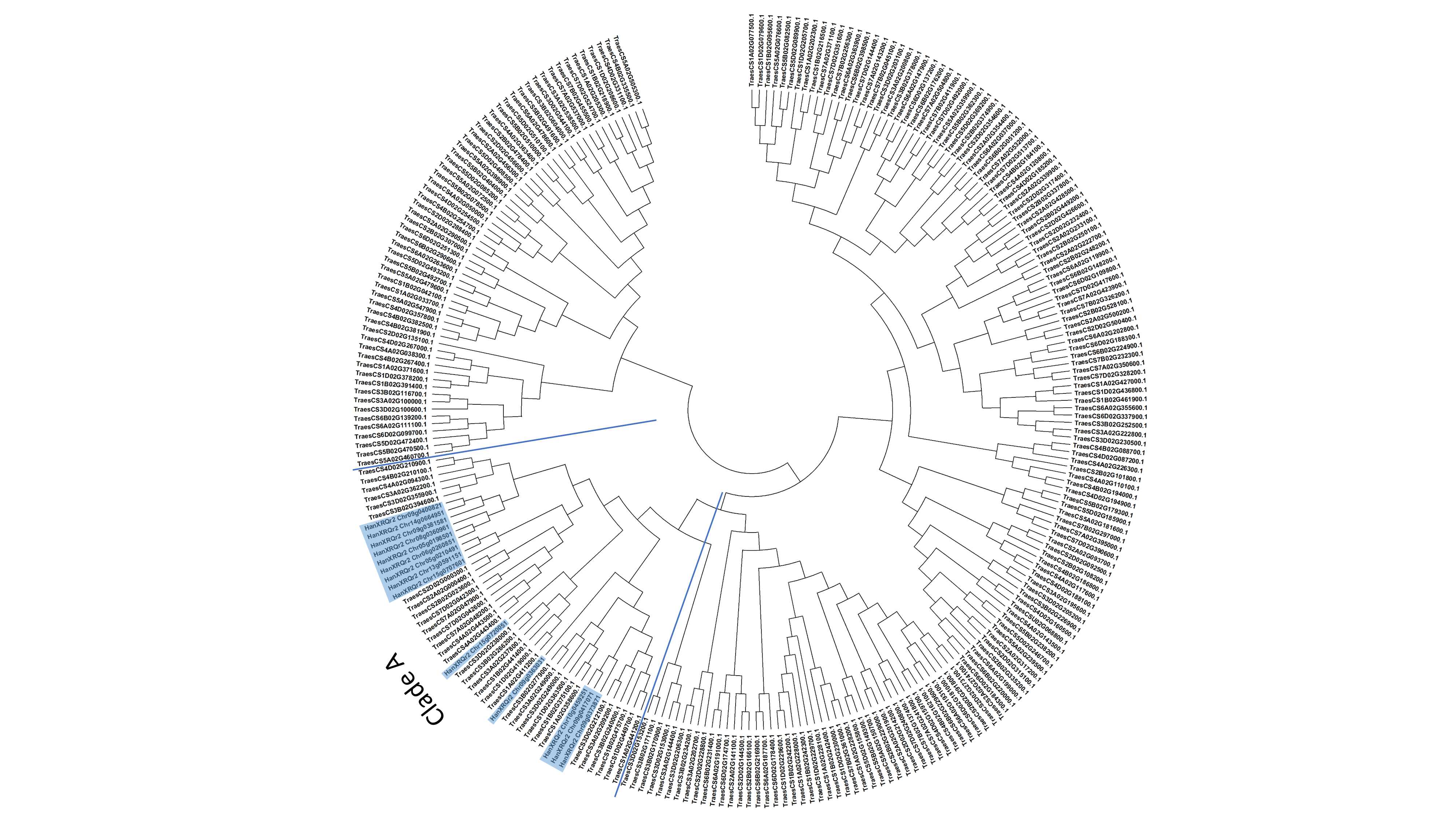

Supplement: Supplementary file 4 [file Image1.tif]
